# diArk – the database for eukaryotic genome and transcriptome assemblies in 2014

**DOI:** 10.1093/nar/gku990

**Published:** 2014-11-06

**Authors:** Martin Kollmar, Lotte Kollmar, Björn Hammesfahr, Dominic Simm

**Affiliations:** Group Systems Biology of Motor Proteins, Department of NMR-based Structural Biology, Max-Planck-Institute for Biophysical Chemistry, Göttingen, 37085, Germany

## Abstract

Eukaryotic genomes are the basis for understanding the complexity of life from populations to the molecular level. Recent technological innovations have revolutionized the speed of data generation enabling the sequencing of eukaryotic genomes and transcriptomes within days. The database diArk (http://www.diark.org) has been developed with the aim to provide access to all available assembled genomes and transcriptomes. In September 2014, diArk contains about 2600 eukaryotes with 6000 genome and transcriptome assemblies, of which 22% are not available via NCBI/ENA/DDBJ. Several indicators for the quality of the assemblies are provided to facilitate their comparison for selecting the most appropriate dataset for further studies. diArk has a user-friendly web interface with extensive options for filtering and browsing the sequenced eukaryotes. In this new version of the database we have also integrated species, for which transcriptome assemblies are available, and we provide more analyses of assemblies.

## INTRODUCTION

Eukaryotic genome research enormously benefits from the increasing number of sequenced organisms. Whereas in the time of Sanger-sequencing single-species analyses and small-scale comparative projects dominated, the throughput of the Illumina technology allowed initiating and conducting the sequencing of thousands of species. Examples are the Genome 10K project (1), the i5k project (2) and the 959 Nematodes project (3) intending to provide the genome sequences of a broad range of species, and the 1001 Arabidopsis project (4), the 1000 bull project (5) and the 3000 rice project (6) aiming to reveal phenotypic and genetic differences of breeds and varieties of economically important animals and plants. Usually, genome assemblies are generated for new species, whereas in population studies the sequencing reads are mapped against reference genomes without producing independent genome assemblies.

NCBI/ENA/DDBJ are the central repositories for sequence read archives (SRAs), the ‘raw data’ for generating assemblies, but publishers and funding agencies often do not require assemblies to also be stored there. Thus, most large-scale sequencing centers like *The Broad Institute of MIT and Harvard* (Cambridge, MA, USA), the *DOE Joint Genome Institute* (Walnut Creek, CA, USA) and *The Wellcome Trust Sanger Institute* (Cambridge, UK) established own species- and taxa-dedicated databases such as Phytozome for plants (7) and the Fungal Genome Initiative project pages (8). Powered by research community efforts, there are also excellent databases dedicated to single species such as FlyBase (9), WormBase (10) and dictyBase (11), or repositories for species of certain taxonomic branches such as EuPathDB (12), VectorBase (13) and FungiDB (14). Although these databases only comprise model species and related organisms, they are well known far beyond their research communities. In contrast, dedicated databases have been set up for many of the newly sequenced species that are only known to small communities. In addition, for many species it takes years from the first release of a draft assembly to the publication of the genome analysis (e.g. the *Babesia bigemina* genome is available since 2003 but was published in 2014; the *Callithrix jacchus* genome has been made available in 2007 but published in 2014). Therefore, it is necessary to have a database to identify and access all the available data.

The two major manually curated genome project databases are GOLD (15) and diArk (16). Whereas GOLD is mainly focused on microbial genomes, we developed diArk as a central hub for all eukaryotes, for which large-scale transcriptome or genome assembly data have been produced and are available to the public. diArk provides measures and analyses of these assemblies, as well as links to the data generator repositories. Currently, diArk comprises 2577 eukaryotes and provides access to almost 6000 transcriptome and genome assemblies.

## DIARK

### Search options and data filtering

diArk provides three main options to search for sequenced eukaryotes. The most straight-forward way to search for a specific species is to use the autocompletion form at diArk's entry page, which is also available in the menu bar and as a browser search plugin. A *FastSearch* allows filtering for taxa, genome type (genome assemblies, EST data, RNA-seq data) and sequencing status (complete and incomplete assemblies), and is currently the most frequently used entry point to diArk. An extended *Search* form provides six search modules that can be combined in any way to search and filter diArk's content. The most widely used modules are the *Taxonomy* module with taxa/species selection integrated into an expandable phylogenetic tree and the *Genome Files* module that provides options to filter for sequencing and assembly methods, genome type, GC content, sequencing coverage and assembly release date. Search options and filters for the new data types generated in the last 3 years have been integrated into the existing modules.

### Result views and statistics

The filtered species can be analysed and compared using seven *Result* tabs. The most frequently used are the pre-selected *Species* tab, the *Genome Files* and the *Sequencing Stats* tab. The *Species* tab provides an overview about species-related information such as alternative, common and anamorph (for many fungi) names, a full taxonomy, a list of all external sources providing access to respective sequence data, and all publications related to the respective species’ sequencing. In the *Genome Files* view, extensive analyses of all assembly files are shown with clickable icons to inspect P50 and A50 plots, CGRs with resolution up to *k* = 10, and details of the used sequencing and assembly methods. All genome assemblies for a given species are listed below each other for fast comparison. While the other *Result* tabs list data species wise, the *Sequencing Stats* view offers many comparisons of the whole selected/filtered species (16).

## CURRENT STATUS OF THE DATABASE

**Figure 1.**
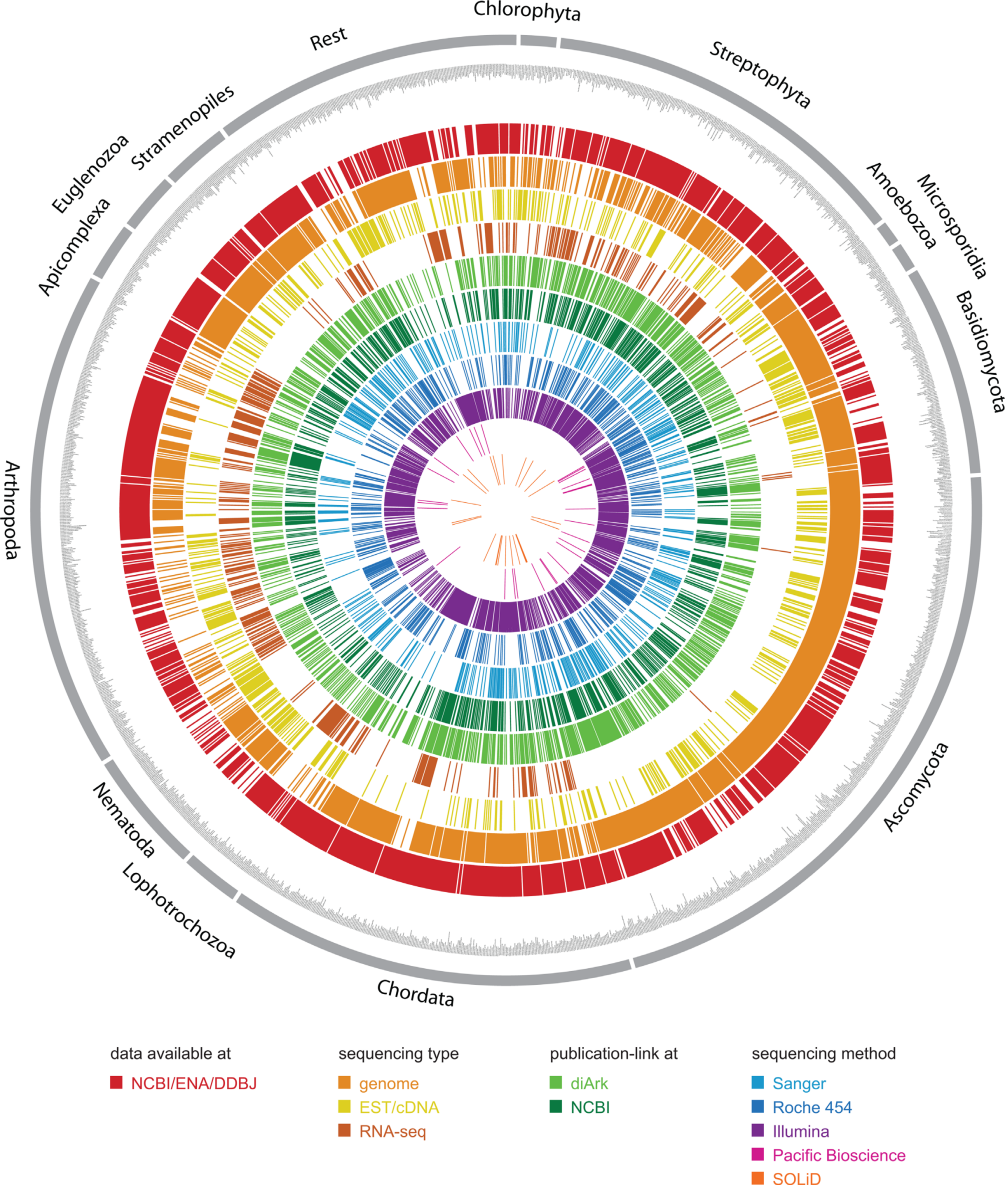
Representation of the nonredundant species (i.e. one strain per species) in diArk with their sequencing type and method. For comparison, all species are marked, for which transcriptome data and/or genome assemblies are available via NCBI/ENA/DDBJ. Nine hundred and eighty five of the assemblies have been published but only 784 of them are linked to the genome assemblies at NCBI.

diArk's growth reflects the exponentially increasing availability of sequenced eukaryotes, now (September 5, 2014) comprising 2577 species (806 in 2011, 415 in 2007). For 1999 of these species (613 in 2011, 209 in 2007) whole genome assembly data are available, and for 429 species transcriptome shotgun assemblies (TSAs; Figure 1), of which the first became available end of 2012. Assembly data for 2017 (78.3%) of the eukaryotes are available at NCBI/ENA/DDBJ meaning that data for 560 (21.7%) species can currently only be accessed at other resources.The data for these 560 species have not yet been or might never be submitted to NCBI/ENA/DDBJ. These species include, for example, the recently published fish *Electrophorus electricus* (17) and the stick insect *Timema cristinae* (18), of which only the SRA data but not the genome assemblies have been deposited at NCBI/ENA/DDBJ, several species such as the snake *Boa constrictor constrictor*, whose genome assemblies are only available at (Giga)^n^DB database (19), and species whose genome assemblies are only available at the sequencing centers such as 31 nematode and 22 Platyhelminth genomes recently finished by *The Wellcome Trust Sanger Institute*. These examples underline the unique value of diArk for the eukaryote sequencing and research community in providing a central hub integrating data available from single species repositories to large-scale sequencing centers. In the last 3 years, RNA-Seq based transcriptome assemblies have essentially replaced EST and cDNA sequencing efforts. Due to the lower costs and faster accessibility, TSAs have almost passed EST/cDNA data in diArk (423 TSAs versus 654 EST/cDNA projects; Figure 1).

diArk links publications of sequence assemblies to species (Figure 1). At NCBI, there is a master record for each assembly to access the respective data, and to which respective publications are linked. Currently, these publication links (784 links) only comprise 79.6% of the publication links included in diArk (985 links). Examples for species, whose genomes have long been published but are still not linked to the NCBI genome entries, include *Ciona savignii* (submitted to NCBI in 2003, published 2007), *Filobasidiella neoformans B-3501A* (submitted 2004, published 2005), *Pristionchus pacificus* (submitted and published 2008), *Culex pipiens quinquefasciatus* (submitted 2007, published 2010) and *Uncinocarpus reesii* (submitted 2005, published 2009). Instead, the genome assemblies of these 200 species are marked as ‘unpublished’. Published and unpublished genomes are important resources for the community but it is also important that data generators get credits for their efforts. On the other hand, embargo rules for unpublished data should indefinitely not prohibit specific analyses (20). At diArk, researchers find the most complete list of references to genome assemblies for proper citation or for selection of appropriate subsets of published species to avoid data usage issues.

In the last years, most of the available genomes have been sequenced with Illumina machines. However, the Sanger method is still used to assist in scaffold and chromosome assembling (Figure 1). Roche's 454 sequencing method is currently the most widely used method for transcriptome shotgun sequencing. Other methods such as SOLiD, PacBio or IonTorrent are still rarely used to generate *de novo* genome or transcriptome assemblies.

## NEW DEVELOPMENTS

**Figure 2.**
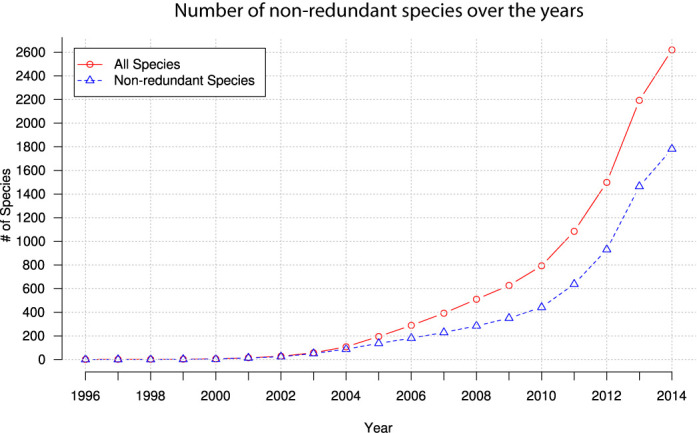
Evolution of the fraction of nonredundant species compared to all sequenced species over time.

diArk hosts and analyses whole-genome and transcriptome assemblies. Currently, the about 6000 assemblies comprise mitochondrial, chloroplast, apicoplast, nucleolar and nuclear genomic DNA and are made available to other services such as the gene reconstruction software WebScipio (21). The quality of genome assemblies can vary significantly (22). However, approaches resulting in excellent genomes for one species might not produce assemblies of similar quality in other cases. Therefore, diArk provides access to alternative assemblies and several measures for direct comparison such as number of contigs, genome size (larger = better), N50 value (higher = better), N50 length (higher = better), contig length distributions (A50 and N50 plots), sequencing coverage (higher = better), sequencing methods and used assembly software. Not only the number of alternative assemblies increased in the last years, but also the number of redundant species in terms of species diversity (Figure 2) increased. Redundant species include, for example, different strains of the same fungal species, different breeds of animals, different varieties of plants and different isolates of protozoa. Within diArk, the respective genome and transcriptome assemblies can directly be compared and the most suitable for a certain research hypothesis be identified. diArk also provides chaos game representations (CGRs), which are fingerprints of genomes, and frequency chaos game representations (FCGRs) at different resolutions, which can be used, for example, for phylogenetic reconstructions (23).

### Integration of RNA-seq data

**Figure 3.**
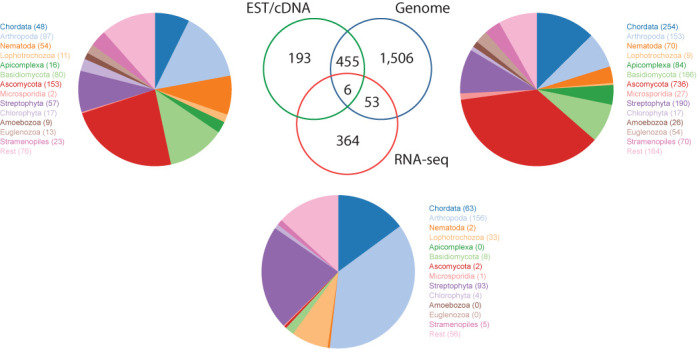
Distribution of species, for which EST/cDNA data, genome assemblies and transcriptome assemblies are available. For each sequencing type, the pie charts show the percentage of sequenced species for selected taxa.

The most noticeable innovation from v.2 to v.3 is diArk's integration of RNA-seq data. The first nonhuman transcriptome assemblies have been submitted to and released by NCBI in late 2012. Since then, not only the diversity of sequenced species has increased rapidly (Figure 3) but also the number of species with transcriptome assemblies generated for different developmental stages and/or organs. Given the low costs of transcriptome compared to genome sequencing, the number of species with available transcriptome assemblies will pass the number of species with sequenced genomes in the near future. Several large-scale projects have already been announced and are expected to release their data this or next year, such as The 1000 plants (oneKP or 1KP) initiative (https://sites.google.com/a/ualberta.ca/onekp/), the Marine Microbial Eukaryote Transcriptome Sequencing project (24) and the Fish-T1K project (http://www.fisht1k.org/). Interestingly, there is not much overlap between species with transcriptome and genome assemblies (Figure 3). One reason is, that RNA-seq data is still rarely generated for species, for which genome assemblies have been produced, and if generated, the RNA-seq data had been used to assist in genome annotation or to generate expression profiles but not to produce independent transcriptome assemblies. In addition, many scientific questions can be answered sufficiently and faster with transcriptome data.

## CONCLUSIONS

Herein, we present an updated version of diArk, which is a central hub for all sequenced eukaryotes, for which either genome or transcriptome assemblies, or large-scale EST/cDNA data are available. diArk is unique in providing direct access to most of the sequenced eukaryotes, whose number has more than tripled compared to the previous version. The number of analysed genome and transcriptome assemblies now reaches 6000.
